# Side effects of omeprazole: a system biology study 

**Published:** 2021

**Authors:** Maryam Hamzeloo-Moghadam, Mostafa Rezaei Tavirani, Somayeh Jahani-Sherafat, Sina Rezaei Tavirani, Somayeh Esmaeili, Mojtaba Ansari, Alireza Ahmadzadeh

**Affiliations:** 1 *Traditional Medicine and Materia Medica Research Center and Department of Traditional Pharmacy, School of Traditional Medicine, Shahid Beheshti University of Medical Sciences, Tehran, Iran*; 2 *Proteomics Research Center, Faculty of Paramedical Sciences, Shahid Beheshti University of Medical Sciences, Tehran, Iran*; 3 *Laser Application in Medical Sciences Research Center, Shahid Beheshti University of Medical Sciences, Tehran, Iran*; 4 *Proteomics Research Center, Shahid Beheshti University of Medical Sciences, Tehran, Iran*

**Keywords:** Omeprazole, System biology, Network analysis

## Abstract

**Aim:**

To assess the effects of omeprazole on the human cardiovascular system is the main aim of this study.

**Background:**

Omeprazole as a proton pump inhibitor is widely consumed to inhibit gastric acid secretion.

**Methods:**

Gene expression profiles of “human coronary artery endothelial cells” in the absence and presence of omeprazole were downloaded from the Gene Expression Omnibus (GEO) database. The differentially expressed genes (DEGs) interacted as an interactome, and the hub nodes are determined. The DEGs were enriched via gene ontology (GO) analysis. The critical hubs were identified based on the GO findings.

**Results:**

Among 103 queried DEGs, 61 individuals were included in the main connected component. CTNNB1, HNRNPA1, SRSF4, TRA2A, SFPQ, and RBM5 genes were identified as critical hub genes. Six clusters of biological terms were introduced as deregulated elements in the presence of omeprazole.

**Conclusion:**

In conclusion, long-term consumption of omeprazole may be accompanied with undesirable effects, however more evidence is required.

## Introduction

 Omeprazole is a proton pump inhibitor applied to inhibit gastric acid secretion (1). There is evidence that omeprazole consumption is accompanied by a few side effects (2). It has been reported that several bone issues occur in patients treated with omeprazole (3). 

High throughput methods such as proteomics, genomics, and metabolomics have attracted the attention of researchers in the fields of medicine and pharmacology and have led to large amounts of data about drugs and their effects on the human body (4). The large amounts of data imply complex analysis to interpret events and produce useful information. PPI network analysis, which is formed based on the interactions between the studied elements, is a suitable method for screening complex sets of data (5). Many diseases are studied using PPI network analysis, resulting in useful information about their molecular mechanisms (6-8). GO analysis also is a suitable method for investigating molecular function, cellular components, biological processes, and biochemical pathways related to the studied genes (9, 10). In the present study the critical deregulated genes of “human coronary artery endothelial cells” in the presence of omeprazole were determined by PPI network analysis, and GO enrichment was applied to the crucial DEGs to achieve a clear understanding of the effects of omeprazole on the human cardiovascular system. 

## Methods

Gene expression profiles of GSE77239/GPL570 were download from GEO (https://www.ncbi.nlm.nih.gov/geo/query/acc.cgi?acc=GSE77239). In this study, the gene expression profiles of 3 cultured “human coronary artery endothelial cells” in the presence of 100 µM omeprazole were compared with cultured cells in the absence of omeprazole as controls (11). The profiles were matched statistically through box plot analysis using GEO2R. The characterized DEGs which had a *p*-value <0.001, adj. *p*-value<0.001, and fold change >2, were identified as significant DEGs. The significant DEGs were included in the PPI network through the STRING (12) database by Cytoscape software (13). As only 37 significant DEGs were included in the main connected component of the constructed network, 20 first neighbor genes from the STRING database were added to the queried DEGs to make a maximum participating number of DEGs in the main connected component. The top 10 nodes (among the queried DEGs) based on degree value were considered hub DEGs.

All significant DEGs were enriched through GO analysis by ClueGO and CluePedia (14), two applications of Cytoscape software. Biological terms were clustered according to a kappa score of 0.5. Term *p-*value, term *p-*value corrected with Bonferroni step down, group *p-v*alue, and group *p-*value corrected with Bonferroni step down were less than 0.001. 

## Results

**Figure 1:**
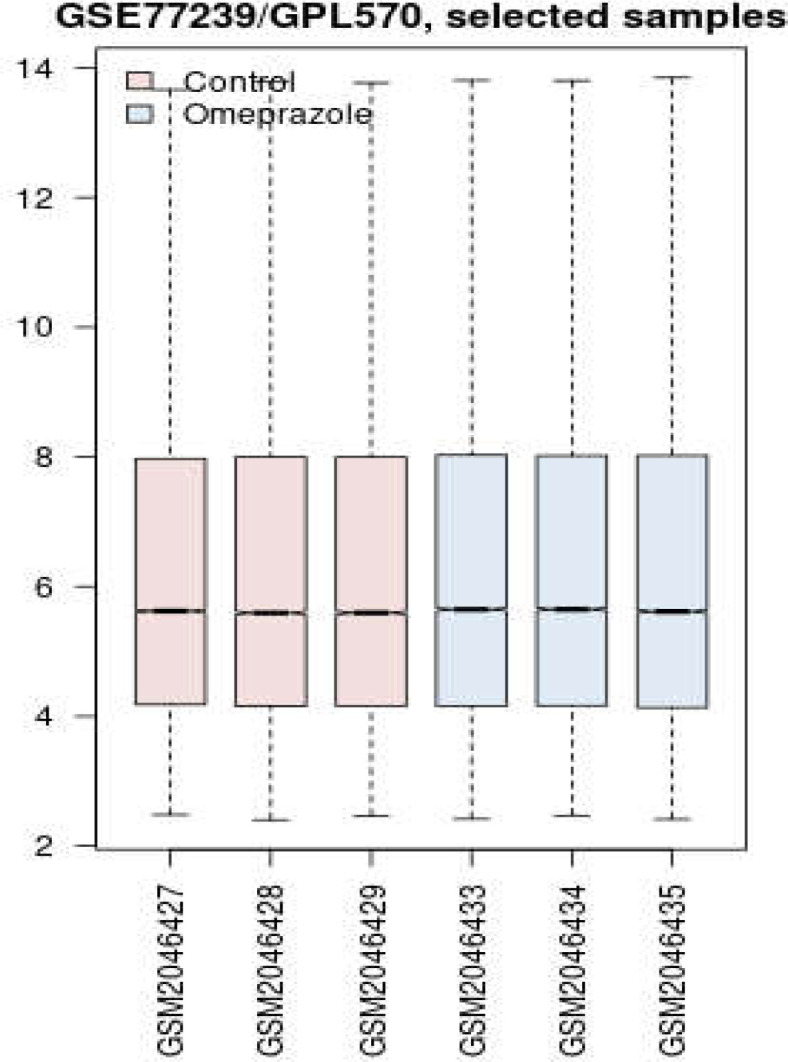
Box plot presentation of gene expression profiles of human coronary artery endothelial cells in the presence and absence of omeprazole

Box plot analysis is shown in Figure 1. GSM2046427-29 as controls and GSM2046433-35 as treated samples were compared. The gene expression profiles are central median and, therefore, statistically comparable.

**Figure 2:**
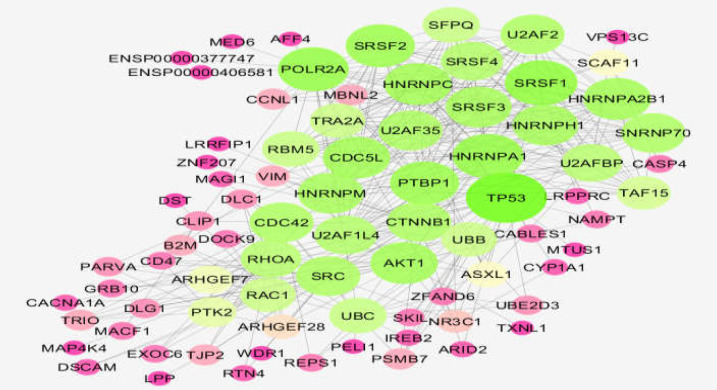
The main connected component including 61 queried DEGs plus 20 first neighbors of human coronary artery endothelial cell PPI network in the presence of omeprazole versus controls. The nodes’ layout is based on degree value. A confidence score of 0.4 is considered

In total, 103 characterized significant DEGs with logFC>2, *p*-value <0.001, and adj. *p*-value <0.001 were included in the PPI network; however, 79 individuals were recognized. The 79 recognized DEGs plus 20 added first neighbors were included in the network. The main connected component comprising 61 queried DEGs (18 DEGs were isolated) plus 20 first neighbors was formed (Figure 2). The 10 hub nodes of the main connected component are shown in table 1.

**Table 1:** Hub nodes of the main connected component. Hubs were selected among the queried DEGs. Descriptions were downloaded from STRING database and are summarized. K refers to degree

R	Name	Description	K
1	HNRNPA1	Heterogeneous nuclear ribonucleoprotein A1; Involved in the packaging of pre-mRNA into hnRNP particles, transport of poly(A) mRNA from the nucleus to the cytoplasm, and may modulate splice site selection.	25
2	CTNNB1	Catenin (cadherin-associated protein), beta 1, 88kDa; Involved in the regulation of cell adhesion. Acts as a negative regulator of centrosome cohesion. Involved in the CDK2/PTPN6/CTNNB1/CEACAM1 pathway of insulin internalization. Blocks anoikis of malignant kidney and intestinal epithelial cells and promotes their anchorage-independent growth. Disrupts PML function and PML-NB formation. Promotes neurogenesis by maintaining sympathetic neuroblasts within the cell cycle.	21
3	U2AF35	U2 small nuclear RNA auxiliary factor 1; Plays a critical role in both constitutive and enhancer-dependent splicing by mediating protein-protein interactions and protein-RNA interactions required for accurate 3'-splice site selection.	21
4	SRSF4	Splicing factor, arginine/serine-rich 4; Plays a role in alternative splice site selection during pre-mRNA splicing. Represses splicing of MAPT/Tau exon 10; RNA binding motif containing.	20
5	U2AF1L4	Splicing factor U2AF 35 kDa subunit-like protein; U2 small nuclear RNA auxiliary factor 1 like 4; RNA binding motif containing.	20
6	U2AFBP	SEE description for U2AF35	19
7	RBM5	Putative tumor suppressor LUCA15; Component of the spliceosome A complex. May both positively and negatively regulate apoptosis.	17
8	SFPQ	Polypyrimidine tract-binding protein-associated-splicing factor; DNA- and RNA binding protein, involved in several nuclear processes. Involved in regulation of signal-induced alternative splicing. During splicing of PTPRC/CD45, a phosphorylated form is sequestered by THRAP3 from the pre-mRNA in resting T-cells; T-cell activation and subsequent reduced phosphorylation is proposed to lead to release from THRAP3, allowing binding to pre-mRNA splicing regulatory elements which repress exon inclusion. Binds the DNA sequence 5'-CTGAGTC-3' in the insulin-like growth factor response element (IGFRE) and inhibits IGF-I-stimulated transcriptional activity. Regulates the circadian clock. Required for the transcriptional repression of circadian target genes. Required for the assembly of nuclear speckles. Plays a role in the regulation of DNA virus-mediated innate immune response.	17
9	TRA2A	Transformer 2 alpha homolog (Drosophila); Sequence-specific RNA-binding protein which participates in the control of pre-mRNA splicing.	16
10	TAF15	TAF15 RNA polymerase II, TATA box binding protein (TBP)-associated factor, 68kDa; RNA and ssDNA-binding protein that may play specific roles during transcription initiation at distinct promoters. Can enter the preinitiation complex together with the RNA polymerase II (Pol II).	15

**Figure 3:**
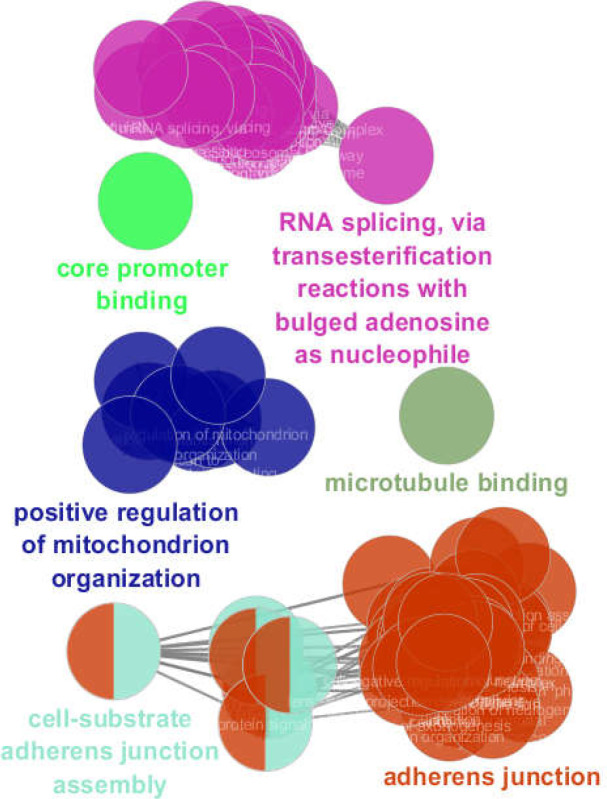
Six clusters of biological terms associated with 103 queried DEGS

**Figure 4:**
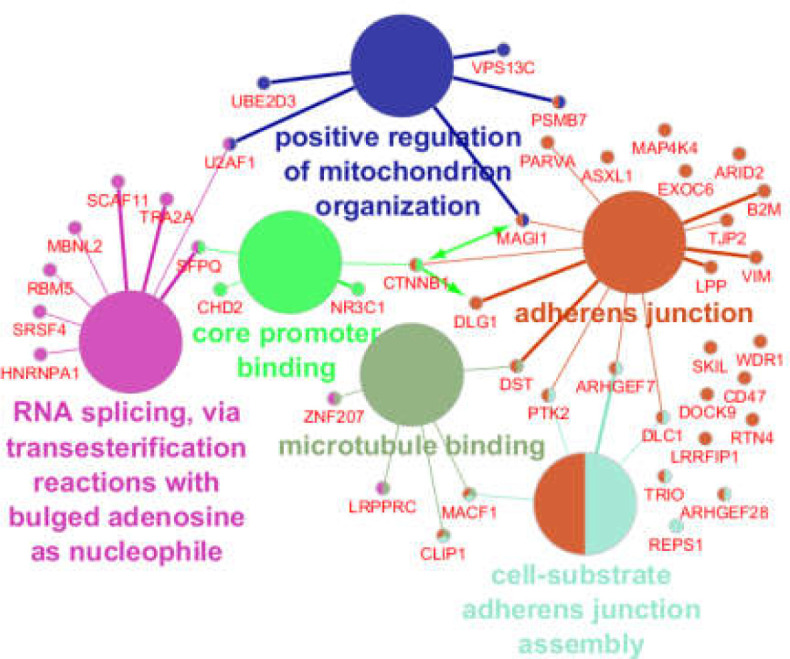
Six clusters of biological terms and associated DEGs. Forty-one of the 103 queried DEGs are related to the clusters. Several DEGs are associated with more than one cluster. The green directed arrow refers to the activation action

GO results of enrichment of the 103 significant DEGs is shown in Figure 3. Six clusters of biological terms including “adherens junction,” “RNA splicing, via transesterification reactions with bulged adenosine as nucleophile,” “positive regulation of mitochondrion organization,” “cell-substrate adherens junction assembly,” “core promoter binding,” and “microtubule binding,” included 37, 20, 8, 6, 1, and 1 terms, respectively. As seen in Figure 4, the “adherens junction” cluster was associated with 26 DEGs (the brown colored genes) while the second cluster; “RNA splicing, via transesterification reactions with bulged adenosine as nucleophile,” was related to 10 genes. The “positive regulation of mitochondrion organization” and “cell-substrate adherens junction assembly” clusters were associated with 5 and 8 DEGs, respectively. 

**Figure 5:**
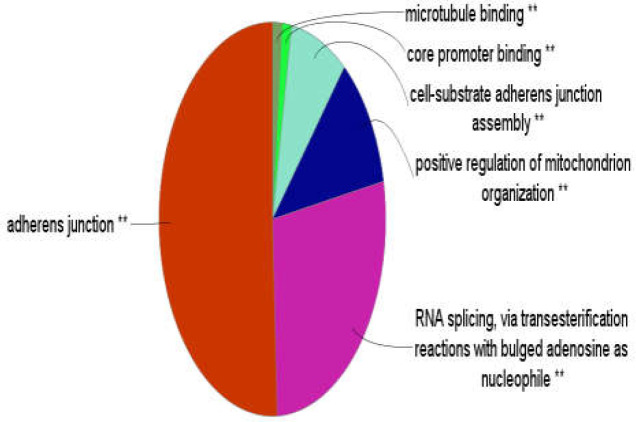
Frequency of biological terms content of the six determined clusters. Group p-values are ≤ 0.01

The fifth and sixth clusters were related to 4 and 5 genes, respectively. Considering the common genes, 41 DEGs among 103 queried DEGs were associated with the biological terms. Figure 5 shows the six clusters identified based on frequency of biological terms content of cluster. As can be seen, “adherens junction” was the largest cluster. Details about the six introduced clusters are presented in table 2.

## Discussion

Network analysis has attracted the attention of researchers in the fields of medicine and pharmacology due to its abilities in discovering hidden features of applications of common drugs. Omeprazole is known as a common drug used to inhibit acid secretion in gastric disorders (15). Yongning Zhouy et al. reported that omeprazole consumption led to the control of bleeding in 59% of patients with portal hypertensive gastropathy (PHG) after 48 h. PHG is an important problem of liver cirrhosis which contributes to acute gastric bleeding (16). In this study, the role of omeprazole on the function of human coronary artery endothelial cells was studied through the assessment of gene expression profiling. The gene expression profiles were statistically matched, and the significant DEGs were included in the PPI network. Ten hub DEGs were identified and six clusters of deregulated biological terms were determined. The largest cluster “adherens junction,” contained 37 biological terms (more than half the total number of terms) is associated with 26 DEGs (63% of the recognized DEGs). The second top hub gene, CTNNB1, was related to the “adherens junction,” while HNRNPA1, SRSF4, TRA2A, SFPQ, and RBM5 were associated with the “RNA splicing, via transesterification reactions with bulged adenosine as nucleophile” cluster. 

**Table 2:** Details of the six introduced clusters of biological terms. Term *p-*value, term *p-*value corrected with Bonferroni step down, group *p-*value, and group *p-*value corrected with Bonferroni step down are less than 0.001. G, R, %AG, NG, and AGF refer to group, row, percentage of associated genes, number of genes, and associated genes found, respectively. Names of clusters (the terms that clusters are called with the name of those terms) are bolded

G		R	GOTerm		Ontology Source	% AG			NG		AGF	
1		1	**Microtubule binding**		GO_MolecularFunction	2			5		[CLIP1, DST, LRPPRC, MACF1, ZNF207]	
2		2	**Core promoter binding**		GO_MolecularFunction	2			4		[CHD2, CTNNB1, NR3C1, SFPQ]	
3		3	Ruffle		GO_CellularComponent	2			4		[ARHGEF7, CLIP1, DLC1, MACF1]	
		4	**Cell-substrate adherens junction assembly**		GO_BiologicalProcess	5			4		[ARHGEF7, DLC1, MACF1, PTK2]	
		5	Focal adhesion assembly		GO_BiologicalProcess	5			4		[ARHGEF7, DLC1, MACF1, PTK2]	
		6	Rho protein signal transduction		GO_BiologicalProcess	2			4		[ARHGEF28, ARHGEF7, DLC1, TRIO]	
		7	Rho GTPase binding		GO_MolecularFunction	2			4		[ARHGEF28, ARHGEF7, REPS1, TRIO]	
		8	Regulation of Rho protein signal transduction		GO_BiologicalProcess	3			4		[ARHGEF28, ARHGEF7, DLC1, TRIO]	
4		9	Regulation of mitochondrion organization		GO_BiologicalProcess	2			5		[MAGI1, PSMB7, U2AF1, UBE2D3, VPS13C]	
		10	**Positive regulation of mitochondrion organization**		GO_BiologicalProcess	3			5		[MAGI1, PSMB7, U2AF1, UBE2D3, VPS13C]	
		11	Positive regulation of establishment of protein localization to mitochondrion		GO_BiologicalProcess	3			4		[MAGI1, PSMB7, U2AF1, UBE2D3]	
		12	Regulation of establishment of protein localization to mitochondrion		GO_BiologicalProcess	3			4		[MAGI1, PSMB7, U2AF1, UBE2D3]	
		13	Protein targeting to mitochondrion		GO_BiologicalProcess	3			4		[MAGI1, PSMB7, U2AF1, UBE2D3]	
		14	Regulation of protein targeting		GO_BiologicalProcess	3			4		[MAGI1, PSMB7, U2AF1, UBE2D3]	
		15	Positive regulation of protein targeting to mitochondrion		GO_BiologicalProcess	4			4		[MAGI1, PSMB7, U2AF1, UBE2D3]	
		16	Regulation of protein targeting to mitochondrion		GO_BiologicalProcess	4			4		[MAGI1, PSMB7, U2AF1, UBE2D3]	
5		17	Spliceosome		KEGG	3			4		[HNRNPA1, SRSF4, TRA2A, U2AF1]	
		18	Formation of Exon Junction Complex		REACTOME_Reactions	3			4		[HNRNPA1, RBM5, SRSF4, U2AF1]	
		19	Formation of the Spliceosomal A Complex		REACTOME_Reactions	4			4		[HNRNPA1, RBM5, SRSF4, U2AF1]	
		20	Formation of the Spliceosomal B Complex		REACTOME_Reactions	3			4		[HNRNPA1, RBM5, SRSF4, U2AF1]	
		21	Formation of an intermediate Spliceosomal C (Bact) complex		REACTOME_Reactions	2			4		[HNRNPA1, RBM5, SRSF4, U2AF1]	
		22	Formation of the active Spliceosomal C (B*) complex		REACTOME_Reactions	3			4		[HNRNPA1, RBM5, SRSF4, U2AF1]	
		23	Lariat Formation and 5'-Splice Site Cleavage		REACTOME_Reactions	3			4		[HNRNPA1, RBM5, SRSF4, U2AF1]	
		24	Cleavage at the 3'-Splice Site and Exon Ligation		REACTOME_Reactions	2			4		[HNRNPA1, RBM5, SRSF4, U2AF1]	
		25	mRNA Splicing - Major Pathway		REACTOME_Pathways	2			4		[HNRNPA1, RBM5, SRSF4, U2AF1]	
		26	mRNA Splicing		REACTOME_Pathways	2			4		[HNRNPA1, RBM5, SRSF4, U2AF1]	
		27	mRNA Processing		WikiPathways	4			5		[HNRNPA1, RBM5, SFPQ, SRSF4, U2AF1]	
		28	Alternative mRNA splicing via spliceosome		GO_BiologicalProcess	7			4		[HNRNPA1, MBNL2, RBM5, SFPQ]	
		29	Splicing		CORUM_CORUM-FunCat-MIPS	2			5		[HNRNPA1, SFPQ, SRSF4, U2AF1, ZNF207]	
		30	mRNA transport		GO_BiologicalProcess	2			4		[HNRNPA1, LRPPRC, SRSF4, U2AF1]	
		31	Regulation of RNA splicing		GO_BiologicalProcess	3			4		[HNRNPA1, MBNL2, RBM5, SRSF4]	
		32	Regulation of mRNA processing		GO_BiologicalProcess	3			4		[HNRNPA1, MBNL2, RBM5, SRSF4]	
		33	RNA splicing via transesterification reactions		GO_BiologicalProcess	2			8		[HNRNPA1, MBNL2, RBM5, SCAF11, SFPQ, SRSF4, TRA2A, U2AF1]	
		34	**RNA splicing via transesterification reactions with bulged adenosine as nucleophile**		GO_BiologicalProcess	2			8		[HNRNPA1, MBNL2, RBM5, SCAF11, SFPQ, SRSF4, TRA2A, U2AF1]	
		35	mRNA splicing via spliceosome		GO_BiologicalProcess	2			8		[HNRNPA1, MBNL2, RBM5, SCAF11, SFPQ, SRSF4, TRA2A, U2AF1]	
		36	Regulation of mRNA splicing via spliceosome		GO_BiologicalProcess	5			4		[HNRNPA1, MBNL2, RBM5, SRSF4]	
6		37	Apoptosis		REACTOME_Pathways	3			5		[CTNNB1, PSMB7, PTK2, TJP2, VIM]	
		38	Apoptotic cleavage of cellular proteins		REACTOME_Pathways	11			4		[CTNNB1, PTK2, TJP2, VIM]	
		39	Cell-Cell communication		REACTOME_Pathways	4			5		[CD47, CTNNB1, DST, PARVA, PTK2]	
		40	Programmed cell death		REACTOME_Pathways	3			5		[CTNNB1, PSMB7, PTK2, TJP2, VIM]	
		42	Primary focal segmental glomerulosclerosis FSGS		WikiPathways	6			4		[CTNNB1, PARVA, PTK2, VIM]	
		43	**Adherens junction**		GO_CellularComponent	2			12		[ARHGEF7, B2M, CTNNB1, DLC1, DLG1, DST, LPP, MAGI1, PARVA, PTK2, TJP2, VIM]	
44	Protein C-terminus binding			GO_MolecularFunction			3	6		[CTNNB1, DLG1, DST, MAGI1, TJP2, VIM]	
45	Cell junction organization			GO_BiologicalProcess			3	8		[ARHGEF7, CTNNB1, DLC1, DLG1, DST, MACF1, PTK2, WDR1]	
46	Apical junction complex			GO_CellularComponent			3	4		[CTNNB1, DLG1, MAGI1, TJP2]	
47	Occluding junction			GO_CellularComponent			3	4		[CTNNB1, DLG1, MAGI1, TJP2]	
48	Cell-substrate adherens junction			GO_CellularComponent			2	9		[ARHGEF7, B2M, CTNNB1, DLC1, DST, LPP, PARVA, PTK2, VIM]	
49	Ruffle			GO_CellularComponent			2	4		[ARHGEF7, CLIP1, DLC1, MACF1]	
50	Bicellular tight junction			GO_CellularComponent			3	4		[CTNNB1, DLG1, MAGI1, TJP2]	
51	Cell-matrix adhesion			GO_BiologicalProcess			2	5		[ARHGEF7, CTNNB1, DLC1, MACF1, PTK2]	
52	Cell junction assembly			GO_BiologicalProcess			4	8		[ARHGEF7, CTNNB1, DLC1, DLG1, DST, MACF1, PTK2, WDR1]	
53	Cell-cell junction organization			GO_BiologicalProcess			3	7		[ARHGEF7, CTNNB1, DLC1, DLG1, MACF1, PTK2, WDR1]	
54	Cadherin binding			GO_MolecularFunction			3	8		[CTNNB1, DLG1, DOCK9, LRRFIP1, MACF1, PARVA, RTN4, TJP2]	
55	Focal adhesion			GO_CellularComponent			2	9		[ARHGEF7, B2M, CTNNB1, DLC1, DST, LPP, PARVA, PTK2, VIM]	
56	Cell-substrate junction assembly			GO_BiologicalProcess			5	5		[ARHGEF7, DLC1, DST, MACF1, PTK2]	
57	Negative regulation of cell development			GO_BiologicalProcess			2	7		[B2M, CTNNB1, MAP4K4, PTK2, RTN4, TJP2, VIM]	
58	Negative regulation of cell projection organization			GO_BiologicalProcess			3	5		[B2M, MAP4K4, PTK2, RTN4, VIM]	
59	Adherens junction organization			GO_BiologicalProcess			4	5		[ARHGEF7, CTNNB1, DLC1, MACF1, PTK2]	
60	Negative regulation of nervous system development			GO_BiologicalProcess			2	6		[B2M, CTNNB1, MAP4K4, PTK2, RTN4, VIM]	
61	Regulation of cell shape			GO_BiologicalProcess			3	5		[DLC1, DLG1, PARVA, PTK2, WDR1]	
62	Heart morphogenesis			GO_BiologicalProcess			3	7		[ARID2, ASXL1, CTNNB1, DLC1, PARVA, PTK2, RTN4]	
63	Cell cortex			GO_CellularComponent			3	7		[ARHGEF7, CTNNB1, DLC1, DST, EXOC6, PTK2, WDR1]	
64	Adherens junction assembly			GO_BiologicalProcess			6	5		[ARHGEF7, CTNNB1, DLC1, MACF1, PTK2]	
65	Cell-substrate adherens junction assembly			GO_BiologicalProcess			5	4		[ARHGEF7, DLC1, MACF1, PTK2]	
66	Negative regulation of neurogenesis			GO_BiologicalProcess			2	6		[B2M, CTNNB1, MAP4K4, PTK2, RTN4, VIM]	
67	Focal adhesion assembly			GO_BiologicalProcess			5	4		[ARHGEF7, DLC1, MACF1, PTK2]	
68	Lens development in camera-type eye			GO_BiologicalProcess			5	4		[CTNNB1, DLG1, SKIL, VIM]	
69	Rho protein signal transduction			GO_BiologicalProcess			2	4		[ARHGEF28, ARHGEF7, DLC1, TRIO]	
70	Negative regulation of neuron differentiation			GO_BiologicalProcess			2	5		[B2M, MAP4K4, PTK2, RTN4, VIM]	
71	Regulation of Rho protein signal transduction			GO_BiologicalProcess			3	4		[ARHGEF28, ARHGEF7, DLC1, TRIO]	
72	Negative regulation of neuron projection development			GO_BiologicalProcess			4	5		[B2M, MAP4K4, PTK2, RTN4, VIM]	
73	Regulation of axonogenesis			GO_BiologicalProcess			3	5		[ARHGEF7, MACF1, PTK2, RTN4, SKIL]	

It can be concluded that both top large clusters and CTNNB1, HNRNPA1, SRSF4, TRA2A, SFPQ, and RBM5 genes are the critical deregulated elements in the presence of omeprazole. 

As shown in table 2, the “adherens junction” cluster contained several essential cellular terms such as apoptosis, cell-cell junction, axonogenesis, neurogenesis, regulation of cell shape, primary focal segmental glomerulosclerosis, and heart morphogenesis. It can be concluded that CTNNB1, which is assigned as a potent hub gene and is linked to the most important cluster of biological terms, is a critical deregulated gene in the presence of omeprazole. Kuechler et al. showed that CTNNB1 is accompanied by intellectual disability (17). Peripheral spasticity, microcephaly, and central hypotonia are highlighted as CTNNB1 mutations (18). Li et al. investigated zebrafish and reported that the overexpression of miR-192 targets CTNNB1 and leads to impaired cardiac development (19). The correlation between CTNNB1 mutation and endometrioid ovarian carcinomas was reported by Palacios et al. (20). As shown in Figure 4, CTNNB1 is connected directly to the “adherens junction” and “core promoter binding” and indirectly to the other 4 clusters. The only activation action for the queried DEGs was found for the MAGI1↔CTNNB1→DLG1 combination. 

Investigation showed that MAGI1 interacts with CTNNB1 to promote cell-cell adhesion structures (21). Moreover, DLG1-PTEN interaction inhibits the axonal stimulation of myelination in Schwann cells (22).

HNRNPA1, SRSF4, TRA2A, SFPQ, and RBM5 are the five hub nodes that are associated with the second large clusters. The term content “RNA splicing via transesterification reactions with bulged adenosine as nucleophile” cluster is mainly involved in the splicing of RNA, a significant process in hypertrophic cardiomyopathy which has been investigated. Ribeiro et al. published a review entitled “RNA Splicing Defects in Hypertrophic Cardiomyopathy: Implications for Diagnosis and Therapy” in 2020. They noted the RNA mis-splicing in hypertrophic cardiomyopathy (23). It seems that the current findings correspond to the published documents, and the identified hub genes can be considered as the valuable affected targets of omeprazole. GO investigation confirmed the results of the PPI network analysis. It can be concluded that the other clusters of biological terms are associated with cardiovascular diseases. The third large cluster is the “positive regulation of mitochondrion organization” individual. This cluster includes terms that are mainly characterized by regulation of the mitochondrion function. There is much evidence for the correlation between deregulation of mitochondrion function and cardiovascular disease (24-26).

At least 6 critical genes, namely CTNNB1, HNRNPA1, SRSF4, TRA2A, SFPQ, and RBM5, and two large clusters of biological terms which are associated with the cardiovascular diseases are deregulated in the presence of omeprazole. It seems that in addition to the advantages of using omeprazole, the side effects of its long-term consumption should be considered to prevent possible damage to the human body; however, more investigations with suitable population sizes are required.
